# The role of self‐criticism and self‐compassion in psychotic experiences and associated distress – Risk or resilience? A systematic literature review

**DOI:** 10.1111/papt.70080

**Published:** 2026-05-24

**Authors:** Megan Crawford, Rachel Terry, Leila Jameel

**Affiliations:** ^1^ Salomons Institute for Applied Psychology, Canterbury Christ Church University Tunbridge Wells UK; ^2^ South London and Maudsley NHS Foundation Trust London UK; ^3^ Centre for Mental Health, Swinburne University of Technology Hawthorn Victoria Australia

**Keywords:** psychosis, resilience, risk, self‐compassion, self‐criticism

## Abstract

**Introduction:**

Research suggests that both self‐criticism and self‐compassion may be associated with psychotic experiences, but the nature of these relationships is unclear. Understanding this further could provide insight into the aetiology of psychotic experiences and support the development of effective interventions. The aim of the current study is therefore to conduct a systematic review of the relationship between self‐criticism, self‐compassion and psychotic experiences (frequency, severity, endorsement and distress) in clinical and non‐clinical populations.

**Method:**

A systematic search of MEDLINE (Ovid), Embase (Ovid) and APA Psycinfo (Ovid) was conducted. Studies were selected according to the inclusion criteria. A quality assessment of eligible studies was conducted.

**Results:**

A narrative synthesis of 17 empirical, quantitative studies revealed high‐quality evidence supporting links between self‐compassion, self‐criticism and a range of psychotic experiences including general, broad measures of overall psychotic experiences; measures of voice‐hearing; measures of unusual beliefs; and measures of paranoia. Self‐criticism appeared most strongly linked to distress associated with psychotic experiences. Longitudinal studies could provide further insight into the role of self‐criticism and self‐compassion in the development of psychosis.

**Conclusions:**

Findings indicate self‐criticism is associated with distressing psychotic experiences, and self‐compassion may be a protective factor, indicating the potential benefits of therapies such as Compassion Focused Therapy.

**Practitioner Points**

- This systematic review explored the relationship between self‐criticism, self‐compassion and psychotic experiences in clinical and non‐clinical populations. It found that self‐criticism is most strongly linked to the distress associated with psychotic experiences, and that self‐compassion may be a protective factor. However, conclusions about the causal relationships between these variables cannot be drawn due to the cross‐sectional designs of included studies.
- The review findings suggest that self‐criticism and self‐compassion are important mechanisms that could be targeted by psychological therapies such as Compassion Focused Therapy (CFT) for psychosis.

## INTRODUCTION

### Psychotic experiences

Psychotic experiences include hearing voices, paranoia, unusual beliefs and speaking and thinking in a disordered manner (Cooke, 2016; Hinterbuchinger & Mossaheb, 2021). Whilst a medical model understands psychosis as a distinct, categorical disorder, the ‘Psychosis Continuum Theory’ views psychotic experiences as existing on a spectrum, suggesting that the severity and impact of these experiences can vary greatly, with some individuals experiencing more frequent, distressing and impairing experiences than others (Kelleher & Cannon, 2011; Van Os et al., 2000, 2009). Whilst psychotic experiences are not distressing for everyone, for many they can be highly distressing and are associated with a decline in social (Palmier‐Claus et al., 2016) and occupational functioning (Fornells‐Ambrojo et al., 2014), suicide and self‐harm (Taylor et al., 2015).

Several cognitive processes are thought to be involved in the onset or maintenance of psychotic experiences, including source monitoring errors (Johns et al., 2001), theory of mind deficits (Healey et al., 2016), jumping to conclusions biases (Dudley et al., 2016) and attributional processes such as external locus of control (Bentall et al., 2001). Recent research has also explored more emotionally charged cognitive processes including guilt (Volpato et al., 2022), shame (Carden et al., 2020), self‐stigma (Dubreucq et al., 2021) and self‐criticism (Waite et al., 2015).

### Self‐criticism and psychosis

Self‐criticism is a transdiagnostic process that appears to be related to a range of psychological difficulties (Gilbert et al., 2001; Gilbert & Irons, 2005). Self‐criticism refers to critical self‐to‐self relating, often accompanied by a sense of disgust and hatred towards oneself (Gilbert et al., 2004). Self‐criticism is often associated with the experience of shame (Gilbert & Irons, 2005) and difficulties with feeling safe and reassured (Gilbert, 2009). High levels of self‐criticism are common for people experiencing psychosis (Gumley et al., 2010) and may be associated with depression (Iqbal et al., 2000), anxiety (Birchwood et al., 2007) and trauma symptoms (Turner et al., 2013). Research indicates that it is appraisals of oneself and of psychotic experiences that lead to distress (Peters et al., 2017), suggesting that self‐criticism may also play a role in the maintenance of psychotic experiences and associated distress.

Self‐criticism has been conceptualised as a counterproductive safety‐seeking strategy which people adopt in response to social threats (i.e., to prevent oneself from appearing or behaving in a way that will result in judgement from others; Gilbert et al., 2004; Gumley et al., 2010; Mayhew & Gilbert, 2008). Social Rank Theory proposes that we have information processing systems that evolved specifically for monitoring dominant‐subordinate social roles and social threat and that when a person has experienced significant hostility or threat, these systems can become more easily activated (Gilbert & Allan, 1998). Social Rank Mechanisms may also play out on a self‐to‐self level, whereby the hostile self threatens the subordinate self (Gilbert et al., 2001). This can further stimulate threat‐related processes such as dissociation, which involves disconnecting from thoughts, feelings, memories or a sense of identity (Gilbert et al., 2001; Stone, 2006). These threat processes appear to overlap with psychotic experiences such as depersonalisation, somatic sensations and sensory intrusions (Gilbert, 2022; Krause‐Utz et al., 2017).

Researchers have conceptualised two aspects of threat in psychosis; one posed by the perceived persecution from others (Heriot‐Maitland et al., 2022), and the other generated by internal negative, critical and hostile voice‐hearing, self‐evaluation and self‐stigma (Birchwood et al., 2000, 2004). Whilst self‐criticism may be adopted as a safety strategy in response to social threat (Gilbert et al., 2012), it can, in turn, be experienced as threatening, leading to negative unintended consequences, including the development and maintenance of psychotic experiences and distress (Martins et al., 2020). A recent quasi‐experimental study investigated whether activating non‐clinical participants' inner critic would affect perception of speech illusions and the emotional valence of perceived words during an auditory perception task (Engeland et al., 2025). The authors found an increase in speech illusions as well as perceived negative tone, suggesting that an activated inner critic may impair source monitoring and shape the content of ensuing auditory experiences.

### Self‐compassion and psychosis

Self‐compassion is defined as “the ability to hold one's feelings of suffering with a sense of warmth, connection and concern” (Neff & McGehee, 2010, p. 226) and appears to be linked to increased wellbeing and reduced distress (Gilbert, 2009; Neff, Kirkpatrick, & Rude, 2007). There is a growing body of evidence that supports the benefits of self‐compassion for subjective wellbeing. Higher levels of self‐compassion have been found to be strongly associated with lower levels of stress, depression and anxiety (MacBeth & Gumley, 2012) and with increased positive affect (Neff, Rude, & Kirkpatrick, 2007). Higher levels of self‐compassion are also reportedly associated with lower rates of positive symptoms of psychosis (Eicher et al., 2013). It has therefore been argued that self‐compassion may be a potent resilience factor for self‐criticism and distress in people with psychosis (Gilbert, 2009; Gumley et al., 2010; Warren et al., 2016). This is supported by a review that indicates that self‐compassion reduces self‐criticism, distress, psychotic symptoms, social marginalisation and improves social functioning (Mavituna et al., 2023).

### Conceptualisation and measurement of self‐compassion and self‐criticism

Although research findings suggest that self‐criticism and self‐compassion are highly related and that self‐compassion may be the antithesis of self‐criticism (Gilbert et al., 2012; Longe et al., 2010), it has been argued that self‐compassion and self‐criticism should be treated as unipolar, semi‐independent constructs to provide more granular insight into protective and risk factors in the context of psychopathology (Ferrari et al., 2022; Muris & Otgaar, 2022). This is supported by evidence that differing neural pathways are involved in self‐compassion and self‐criticism (Brenner et al., 2017). For example, Longe et al. (2010) functional magnetic resonance imaging (fMRI) study found self‐reassurance was linked to activation of the dorsolateral prefrontal cortex, left temporal pole and insula, whereas self‐criticism was linked to activation in the ventrolateral prefrontal cortex and dorsal anterior cingulate. Similarly, Kim et al.'s (2020) fMRI study demonstrated differences within the threat system, with significantly greater neural activation in the amygdala when participants were self‐critical versus self‐reassuring.

### Aim of current review

This review aims to synthesise the current evidence base regarding the relationship between self‐criticism, self‐compassion and psychotic experiences (including measures of frequency, severity, distress and endorsement). In line with dimensional approaches to psychosis (Kelleher & Cannon, 2011; Van Os et al., 2000), this review will include participants with a broad range of psychotic experiences along a continuum of severity, distress and impact, who may or may not have a formal psychiatric diagnosis. This review will distinguish between studies that treat self‐criticism and self‐compassion as unipolar constructs and those that do not, in order to provide further insight into the link with vulnerability and resilience to psychosis. Thus, this review seeks to answer the following questions:

1. How do self‐criticism and self‐compassion relate to different types of psychotic experiences?

2. How do self‐criticism and self‐compassion relate to frequency, severity, distress or endorsement of psychotic experiences?

## METHOD

### Inclusion and exclusion criteria

As per the aims of this review, papers were identified that explored the relationship between self‐compassion/self‐criticism and psychotic experiences. Empirical studies were included if they met the following criteria: (1) were written in English; (2) included a quantitative measure of self‐criticism or self‐compassion; (3) included a measure of psychotic/psychotic‐like symptoms; (4) specifically examined the relationship between self‐criticism/self‐compassion and psychotic/psychotic‐like symptoms. Unpublished/grey literature was not included.

### Measures of self‐compassion and self‐criticism

The most commonly used measures of self‐criticism and self‐compassion are Forms of Self‐Criticising/Attacking and Self‐Reassuring Scale (FSCRS; Gilbert et al., 2004), The Functions of Self‐Criticism/Self‐Attacking Scale (FSCS; Gilbert et al., 2004) and The Self‐Compassion Scale (SCS; Neff, 2003a, 2003b). All of the studies in the review utilised one or more of these measures, though this was not a prerequisite for inclusion. The FSCRS captures different forms of self‐criticism and self‐reassurance, and the FSCS is used to distinguish between functions of self‐criticism (see Table 1). Some studies reported independent subscale scores of the FSCS and FSCRS, whilst some reported combined scale scores, both of which were eligible for inclusion. The Self‐Compassion Scale (SCS) measures the three main components of self‐compassion (see Table 1).

**TABLE 1:** Table outlining the subscales of the FSCRS, FSCS and SCS.

Scale	Subscales	Description
Forms of Self‐Criticising/Attacking and Self‐Reassuring Scale (FSCRS)	Hated Self	Captures a more destructive, disgust‐based response to setbacks (rather than feelings of inadequacy) characterised by self‐hate and a persecutory desire to hurt the self.
	Inadequate Self	Captures a sense of feeling internally put‐down and feelings of inadequacy linked to perceived failures and setbacks.
	Reassured self	Reassured self focuses on the sense of being able to forgive, support and show care towards oneself, which is similar to self‐compassion.
Function of Self‐Criticising/Self‐Attacking Scale (FSCS)	Self‐persecutory	Self‐criticism that involves expressing anger towards oneself with the intention of wanting to harm oneself.
	Self‐correction	A type of self‐criticism that is more likely to be used for self‐improvement and to stop the self from making mistakes.
Self‐Compassion Scale (SCS)	Self‐kindness vs. self‐judgement	Responding to oneself in a warm and understanding manner when we suffer, fail or feel inadequate, rather than being self‐critical, judgmental and or angry with oneself (Neff, 2003a).
	Common humanity vs. isolation	The ability to recognise that challenges, suffering and flaws are a common human experience, rather than feeling isolated and alone with ones' experiences.
	Mindfulness vs. over‐identification	Being able to hold suffering in mindful awareness without over‐identifying or becoming fused with it.

It has been argued that the use of the SCS Total Score as an individual index of self‐compassion is problematic as the negatively worded items of SCS appear to relate more to self‐criticism than self‐compassion (López et al., 2015; Muris & Petrocchi, 2017). Thus, studies vary in whether they use the one‐factor SCS, the two‐factor SCS, or the six‐factor SCS (see Table 2). Studies using any of the three variants of the SCS were included in the review to allow for comparison of how different factor structures may relate differentially to psychotic experiences.

**TABLE 2:** Table outlining the one‐factor, two‐factor and six‐factor models of the SCS.

Factor model	Scoring
One‐factor SCS	The three positively worded items (self‐kindness, common humanity, mindfulness) and three negatively worded items (self‐judgement, isolation, over‐identification) are all scored together to produce an overall score of self‐compassion (often referred to as total self‐compassion).
Two‐factor SCS	The three positive worded items (self‐kindness, common, humanity, mindfulness) are scored together to produce a score for the positive aspects of self‐compassion (often referred to as positive self‐compassion subscale). The three negatively worded items (self‐judgement, isolation, over‐identification) are scored together to produce a score for the negative aspects of self‐compassion/self‐criticism (often referred to as negative self‐compassion) subscale.
Six‐factor SCS	All six items are scored independently to produce six scores capturing the different aspects of self‐compassion/self‐criticism.

### Search strategy

This review was conducted according to the Preferred Reporting Items for Systematic Reviews and Meta‐Analyses (PRISMA) guidelines (Moher et al., 2010), and a systematic review protocol was registered with PROSPERO in advance (PROSPERO registration: CRD42024591652). A systematic search of MEDLINE (Ovid), Embase (Ovid) and APA Psycinfo (OVID) was first conducted on 10/01/2025. The search was repeated on 08/09/2025 to check for new publications, and the PRISMA flow diagram was updated accordingly (see Figure 1). Key search terms included psychosis, unusual experiences, hallucinations, voice‐hearing, delusions, unusual beliefs, paranoia, self‐criticism and self‐compassion, reflecting terms used in previously published reviews on the role of self‐compassion and self‐criticism in psychopathology (MacBeth & Gumley, 2012; Muris & Petrocchi, 2017; Werner et al., 2019), as well as reviews on the role of shame (Carden et al., 2020) and guilt (Volpato et al., 2022) in psychosis. Reference lists of identified papers were also searched to further identify relevant literature. Parallel screening and in‐depth assessment of eligibility were undertaken by a second reviewer (RT), and there was 100% level of agreement between reviewers at the end of this process.

**FIGURE 1:**
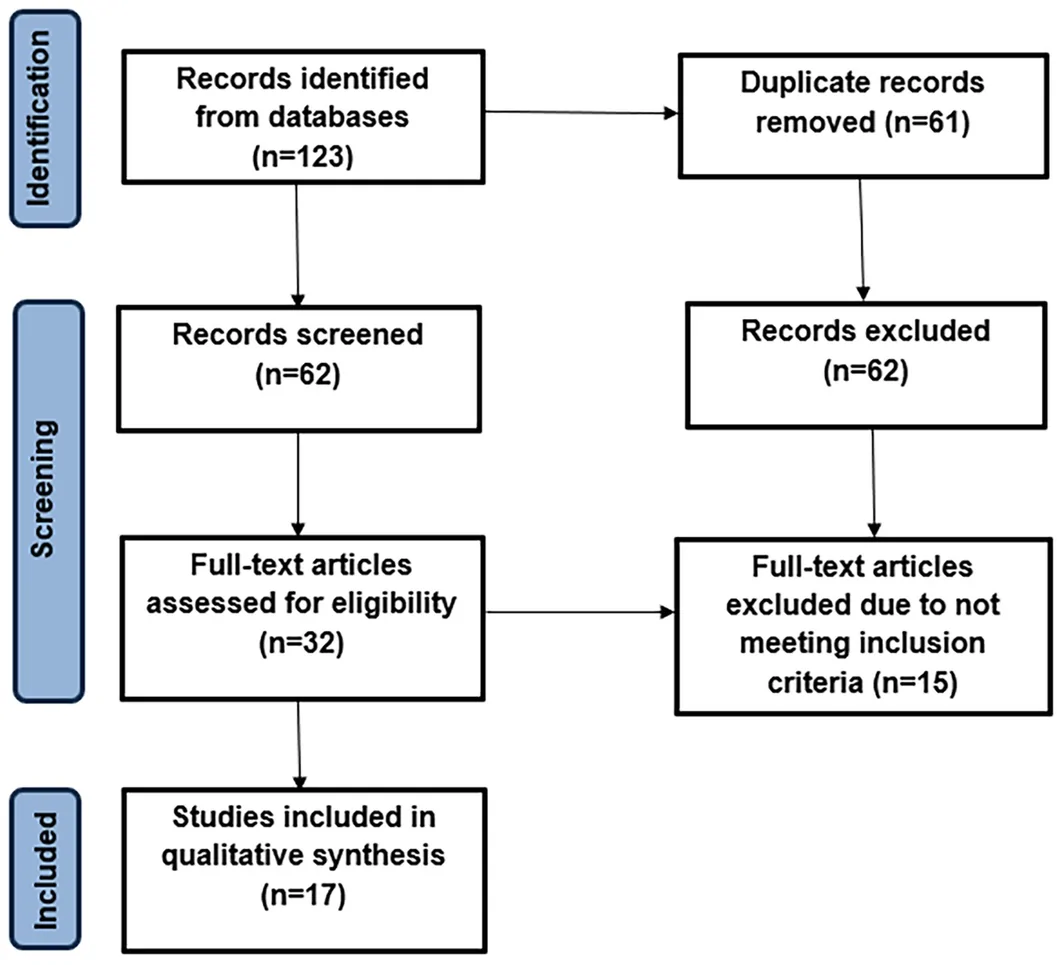
PRISMA (2009) Flow Diagram showing stages of screening and exclusion of studies.

### Data extraction and analytic plan

Data was extracted by the lead author using an extraction spreadsheet. As meta‐analysis of the data was not possible due to the heterogeneity of samples, measures and analysis (Lau et al., 1997), a narrative synthesis was conducted, which is commonly used where it is not appropriate or possible to meta‐analyse the data (Campbell et al., 2018). Structured tabulation of the data was completed to aid transparency between review‐level data and conclusions (Popay et al., 2006; Table 3). Extracted data included study design, sample characteristics, measure of psychotic experiences, measure of self‐criticism or self‐compassion, analysis and findings, which were in line with a similar previous systematic review (Carden et al., 2020).

**TABLE 3:** Table of included studies and relevant extracted data.

Author, date	Design	Sample	Diagnosis and diagnostic tool	Measurement of psychosis experiences	Measurement of self‐compassion	Measurement of self‐criticism	Main (relevant) findings
Böge et al. (2022)	Cross‐sectional	79 participants. 54 male, 24 female, 1 diverse. (M = 42.29, SD = 13.41). 87.34% German nationality, 2.53% Turkish nationality, 10.13% other.	ICD‐10. Affective and non‐affective psychosis.	Positive and negative symptoms using PANSS and SNS.	Six‐factor SCS	N/A	Only the self‐judgement subscale of the SCS was positively correlated with the PANSS negative scale (*r* = 0.54). SCS total scale was significantly associated with the SNS (*r* = −0.37), as were the negatively formulated subscales of self‐judgement (*r* = 0.39), isolation (*r* = 0.48) and overidentification (*r* = 0.28). SCS total score moderated the negative relationship between mindfulness and cognitive fusion (*b* = −0.50) and significantly moderated the indirect effect of mindfulness and cognitive fusion on SNS (CI [0.01, 0.15]).
Carvalho et al. (2019)	Cross‐sectional	1657 participants. 870 female, 787 male (M = 16.47, SD = 1.04). Ethnicity not reported.	No diagnosis.	Paranoia using the Paranoid ideation scale	N/A.	FSCRS	Self‐criticism correlated with Persecutory Ideas (*r* = .543) Self‐deprecation (r = .503) and Mistrust Feelings (*r* = .393). For persecutory ideas External shame was the strongest predictor followed by self‐criticism, and for mistrust feelings and self‐deprecation external shame was the strongest predictor followed by shame experiences and self‐criticism.
Cheli et al. (2023)	Cross‐sectional study	2065 participants. 65.1% female, 34.9% male (M = 22.09, SD = 3.33) Ethnicity not reported.	No diagnosis.	Delusions using the PDI.	N/A	FSCRS – self‐hate subscale	Delusional ideation correlated with self‐criticism (*r* = 0.367). Self‐criticism predicted delusional ideation (*b* = 0.309), and significantly moderated the relationship between self‐reflectivity and delusional ideation (*R* ^2^ = 0.1946; *F* = 9.8605; *p* = .0000).
Collman et al. (2023)	Cross‐sectional study	531 participants. 80% female, 20% male. 69.9% aged between 18 and 29. 45.2% White British, 30% White Other, 6.2% Mixed heritage, 13.9% Asian, 1.5% Black, 3.2% Other.	No diagnosis.	Psychotic like experiences using the TEQ.	Two‐factor SCS‐short form	FSCRS.	TEQ positively correlated with FCRS‐HS (*r* = 0.240), FCRS‐IS (0.167) and negative SCS subscales (*r* = 0.794). TEQ was not correlated with the positive SCS subscales (*r* = −0.035). Only higher FSCRS‐HS scores predicted higher TEQ scores (*B* = 0.15).
Connor and Birchwood (2013)	Cross‐sectional study	74 participants. 40.5% female, 59.5% male (M = 43, SD/range not reported). 64.9% White European, 20.3% Afro‐Caribbean, 6.8% Indian‐Asian, 4.1% mixed heritage, 2.7% British Pakistani, 1.4% African.	Diagnostic tool not specified. Affective and Non‐affective psychosis.	Emotional expression and power of voices using the SCID, VPD and LEE.	N/A	FSCRS a.	Emotional expression of voices was predicted by FSCR inadequate self (*b* = 0.478). Power of voices was predicted by FSCRS hated self (*b* = 0.38). Affiliative themes of voice content were negatively predicted by FSCS self‐persecution (*b* = −0.308). Shaming theme of voice content was negatively predicted by the FSCRS self‐reassuring scale (*b* = −0.273).
Dudley et al. (2018)	Cross‐sectional study	128 participants. 73% female, 27% male (M = 37.5, range 18–74). 87.5% Caucasian, 2.3% Latin/Hispanic, 0.8% Middle Eastern, 0.8% African, 1.6% Asian, 3.1% mixed heritage, 3.1% other.	ICD‐10. 107 participants with affective and non‐affective psychosis. 21 participants with no diagnosis.	HPSVQ	One‐factor SCS	N/A	Self‐compassion correlated with voice severity (*r* = −0.51) distress (*r* = −0.45), and mindfulness of voices (*r* = 0.58). Self‐compassion mediated the relationship between mindfulness of voices and severity of voices (CI[−0.14, 0.00]), and mindfulness of voices significantly mediated the relationship between self‐compassion and severity of voices (CI [2.19, 1.10]).
Eicher et al. (2013)	Cross‐sectional study	88 participants. 12 women, 76 men (M = 49.61, SD = 8.40). 52.27% African American, 46.59% Caucasian, 1.14% other.	SCID. Affective and non‐affective psychosis.	PANSS	One‐factor and six‐factor SCS	N/A	PANSS positive scores correlated with total SC (*r* = −0.33) and SC subscales of self‐judgement (*r* = 0.27), isolation (*r* = 0.30) and overidentification (*r* = 0.23). PANSS excitement scores correlated with total SC (−0.26) and subscales of self‐kindness (*r* = −0.32), common humanity (*r* = −0.35) and mindfulness (*r* = −0.37). PANSS emotional discomfort scores were correlated with total SC (*r* = −0.48) and subscales of mindfulness (*r* = −0.23) self‐judgement (*r* = 0.48), isolation (*r* = 0.42) and overidentification (*r* = 0.42). Only the individual PANSS items of depression (*B* = −0.44) and hostility (*B* = 0.25) were found to predict lower total self‐compassion. There was still a trend between PANSS positive and SCS total scores (*B* = −0.19) when controlling for the individual PANSS item of depression.
Hardman et al. (2023)	Cross‐sectional	145 participants. 66 female, 79 male (M = 20.81, SD = 2.85). Ethnicity not reported.	DSM‐IV. Affective and non‐affective psychosis.	PANSS	One‐factor and two‐factor SCS‐ short form	N/A	Negative symptoms correlated with the negative self‐compassion subscale (*r* = 0.167) and self‐compassion total score (*r* = −0.197). Negative symptoms were not correlated with the positive self‐compassion subscale.
Hutton et al. (2013)	Case–control	49 participants. Persecutory delusion group: 11 female, 4 male (M = 38.5, SD = 13.1). Depression group: 9 female, 6 male (M 39.3, SD 13.7). Control group: 11 female, 8 male (M = 28, SD = 9.8). Ethnicity not reported.	Diagnostic tool not specified. Affective and non‐affective psychosis.	Delusions and paranoia using the PSYRATS–delusion subscale and PANSS‐suspiciousness subscale.	N/A	FSCRS and FSCS.	Greater hated‐self (*U* = 52.5) and inadequate self (*U* = 69) and lower self‐reassurance (*U* = 20) in the unusual beliefs group compared to healthy controls No differences in self‐criticism between the unusual beliefs group and the depression group. Lower self‐corrective self‐criticism in unusual beliefs compared to healthy controls (*p* = .012, *d* = −1.21). No differences in persecutory self‐criticism between groups. Self‐persecutory self‐attacking was significantly associated with paranoia across the groups. Associations between paranoia and inadequate self‐criticism, hated self‐attacking and reduced self‐reassurance disappeared after controlling for depression. No association between self‐corrective self‐criticism and either paranoia or depression was observed.
Hochheiser et al. (2020)	Cross‐sectional study	92 participants. 13 female, 79 male (M = 49.42, SD = 8.6). 53% African American, 46% White, 1% Other.	DSM‐IV. Affective and non‐affective psychosis.	PANSS	Two‐factor SCS	N/A	PANSS scores were correlated with SCS‐positive (*r* = −0.23) and SCS‐negative (*r* = 0.24). Relationships not significant in regression analysis.
Kingston and Schuurmans‐Stekhoven (2016)	Cross‐sectional	250 participants. 79.6% female, 20.4% male (M = 35.72, SD = 9.74) Ethnicity not reported.	No diagnosis.	Delusions using the PDI.	N/A	FSCRS.	PDI correlated with self‐criticism (*r* = 0.46) and self‐reassurance (*r* = −0.27). Self‐criticism but not self‐reassurance was found to mediate the relationship between life hassles and delusional ideation (*R* ^2^ = 0.27, *F* = 22.96).
Maisey et al. (2022)	Cross‐sectional study	62 participants. 39 female, 22 male. (M = 35, SD = = 12.3). Ethnicity not reported.	Diagnostic tool not specified. affective and non‐affective psychosis, PTSD and other non‐specified diagnosis. 4 participants no diagnosis.	Voice hearing using PSYRATS – hallucinations subscale.	One‐factor SCS‐short form	N/A	Self‐compassion correlated with voice distress (*r* = −0.51) and PTS symptom severity (*r* = −0.45) but not with voice frequency (*r* = −0.22). Self‐compassion predicted voice distress (*β* = −0.39) but did not moderate the relationship between PTS and voice distress.
Mills et al. (2007)	Cross‐sectional study.	131 participants. 83 females, 48 males (M = 22.10, SD = 6.0). Ethnicity not reported.	No diagnosis.	Paranoia using the Paranoid ideation scale	Six‐factor SCS	FSCRS and FSCS.	Paranoid ideation positively correlated with criticism subscales of inadequate self (*r* = 0.32), hated self (*r* = 0.44), self‐correction (*r* = 0.33), self‐persecution (*r* = 0.40) and self‐compassion subscales of self‐judgement (*r* = 0.20), isolation (*r* = 0.25) and overidentification (*r* = 0.17). Paranoid ideation negatively correlated with the criticism subscale of reassured self (*r* = −0.27), and self‐compassion subscale of self‐kindness (*r* = 0.23). Self‐hating was found to be the most important predictor or paranoia (*b* = 0.33), followed by depression (*b* = 0.23).
Norman et al. (2020)	Cross‐sectional study	53 participants. 30 female, 23 male (M = 34.62, SD = 12.98). Ethnicity not reported.	Diagnostic tool not specified. Affective and non‐affective psychosis, other non‐specified diagnosis.	Voice‐hearing using PSYRATS – Hallucinations subscale and BVQ.	One‐factor SCS‐short form	N/A	Self‐compassion was negatively correlated with voice distress (*r* = −0.40), voice omnipotence (*r* = −0.34) and voice malevolence (*r* = −0.44). Self‐compassion was found to significantly predict depression (*β* = −.33), malevolence (*β* = −0.54), and voice distress (*β* = −0.39).
Pinto‐Gouveia et al. (2013)	Cross‐sectional study	204 participants. 144 female, 60 male (M = 36.06, SD = 10.22). Ethnicity not reported.	No diagnosis.	Paranoia using Paranoid Ideation Scale.	N/A	FSCRS and FSCS. Self‐Criticism.	The self‐criticism subscale (hated self plus inadequate self) of the FSCRS was positively correlated with paranoia (*r* = 0.48), but the reassuring subscale was not. The FSCS (self‐correction plus self‐persecution) were also positively correlated with paranoia (*r* = 0.38). Paranoia was predicted by self‐criticism with a self‐persecutory function, though self‐criticism did not mediate the relationship between shame memories and paranoia.
Richardson et al. (2023)	Case–control study	Clinical group: 55 participants. 33 male, 22 female. (M = 40.22, SD = 13.06). 87.3% White, 5.5% Black, 1.8% Asian, 3.6% mixed ethnicity, 1.8% other. Non‐clinical group: 166 participants. 120 female, 45 male, 1 non‐binary (M = 21, SD = 4.79). 79.5% White, 2.4% Black, 7.2% Asian, 6.6% mixed ethnicity, 4.2% other.	DSM‐IV. Affective and non‐affective psychosis.	Positive and negative symptoms using Prodromal Questionnaire – Brief version.	One‐factor SCS – short form	N/A	Clinical group had a higher score of childhood sexual abuse (*F* = 4.47), positive symptoms (*F* = 54.3), distress (*F* = 66.82) and paranoia (*F* = 60.05). There was no between‐group difference in self‐compassion (*F* = .58). In both groups, lower‐self‐compassion mediated the relationship between CSA and paranoia (non‐clinical: CI = [0.26, 1.96]; clinical: CI = [0.01, 5.13]). In the clinical group, lower levels of self‐compassion predicted positive symptoms (*b* = −0.24). In the non‐clinical group but not the clinical group, self‐compassion mediated the relationship between CSA and positive symptoms (CI = [0.04, 0.45]) and between CSA and distress (CI = [0.18, 1.75]).
Scheunemann et al. (2019)	Cross‐sectional study	234 participants. 62.4% female, 33.3% male (M = 37, SD = 15). Ethnicity not reported.	No diagnosis.	Delusions and voice‐hearing using the PDI and LSHS.	One‐factor and six‐factor SCS	N/A	Lower scores in self‐compassion (total score and all its facets) were associated with a higher frequency of delusional experiences (*r* = 0.27) and hallucinatory experiences (*r* = 0.26), and negatively correlated with delusional distress (*r* = −0.37) and hallucinatory distress (*r* = −0.37). Self‐compassion correlated significantly more with distress than with frequency for unusual beliefs (*Z* = 3.71) and hallucinations (*Z* = 2.38). This pattern was also seen for facets of self‐kindness, self‐judgement, isolation, and over‐identification. A network model revealed that the shortest pathway between PLE‐ dimensions and self‐compassion facets was between isolation and hallucination and delusion distress.

Abbreviations: BVQ, Beliefs About Voices Questionnaire (Chadwick et al., 2000); DSM, Diagnostic and Statistical Manual of Mental Disorders (American Psychiatric Association); FSCRS, Forms of Self‐Criticising/Attacking and Self‐ Reassuring Scale (Gilbert et al., 2004); FSCS, The Functions of Self‐Criticism/Self‐Attacking Scale (Gilbert et al., 2004); HPSVQ, The Hamilton Program for Schizophrenia Voices Questionnaire (Van Lieshout & Goldberg, 2007); ICD, International Classification of Diseases (World Health Organisation, 1993); LEE, The Level of Expressed Emotion Scale (Cole & Kazarian, 1988); PANSS, Positive and Negative Symptom Scale (Kay et al., 1987); PDI, Peters' Delusion Inventory (Peters et al., 2004); PSYRATS, Psychotic Symptom Rating Scales (Haddock et al., 1999); SCID, Structured Clinical Interview for DSM Disorders (Williams et al., 2015); SCS, Self‐Compassion Scale (Neff, 2003a, 2003b); SNS, Self‐Evaluation of Negative Symptoms (Dollfus et al., 2016); TEQ, Transpersonal Experiences Questionnaire (Heriot‐Maitland et al., 2023); VPD, The Voice Power Differential Scale (Birchwood et al., 2004).

### Risk of bias

Risk of bias was assessed using The Agency for Healthcare Research and Quality Risk of Bias Tool (Williams et al., 2010). This tool covers 11 methodological domains, and criteria can be specifically adapted for the context of the review. This tool was chosen as it has been used for reviews of observational data in a variety of contexts (Carden et al., 2020; Dudley et al., 2016; Taylor et al., 2015). Risk of bias assessments were undertaken by the first and second authors. The final level of agreement for both quality assessment tools was 100% (see Table 4).

**TABLE 4:** Risk of Bias Assessment.

Author	Selection bias	Comparison group	Sample size	Description of cohort	Measures of self‐compassion/self‐criticism	Measures of psychotic symptoms	Blinding	Missing data?	Confounding variables	Analytic methods
Böge et al. (2022)	Yes	Not applicable	Yes	Yes	Yes	Yes	Cannot tell	Cannot tell	Yes	Yes
Carvalho et al. (2019)	Partially	Not applicable	No	Yes	Yes	Yes	Cannot tell	Cannot tell	Yes	Yes
Cheli et al. (2023)	Yes.	Not applicable	No	Yes	Yes	Yes	Cannot tell	No	Yes	Yes
Collman et al. (2023)	Yes	Not applicable	No	Yes	Yes	Yes	Yes	Cannot tell	Yes	Yes
Connor & Birchwood (2013)	Yes	Not applicable	No	Yes	Yes	Partially	Cannot tell	Cannot tell	No	Yes
Dudley et al. (2018)	Partially	Not applicable	Yes	Yes	Yes	Yes	Yes	Yes	No	Yes
Eicher et al. (2013)	Partially	Not applicable	No	Yes	Yes	Yes	Cannot tell	Cannot tell.	Yes	Yes
Hardman et al. (2023)	Yes	Not applicable	No	Yes	Yes	Yes	Yes	No	Yes	Yes
Hochheiser et al. (2020)	Partially	Not applicable	No	Partially	Yes	Yes	Cannot tell	Cannot tell	Yes	Yes
Hutton et al. (2013)	Yes	Yes	Yes	Yes	Yes	Yes	Cannot tell	No	Yes	Yes
Kingston & Schuurmans‐Stekhoven (2016)	Partially	Not applicable	Yes	Yes	Yes	Yes	Yes	No	Yes	Yes
Maisey et al. (2022)	Yes	Not applicable	No	Yes	Yes	Partially	Yes	Cannot tell	No	Yes
Mills et al. (2007)	Partially	Not applicable	No	No	Yes	Yes	Can't tell	Can't tell	No	Yes
Norman et al. (2020)	Yes	Not applicable	Yes	Yes	Yes	Partially	Yes	No	Yes	Yes
Pinto‐Gouveia et al. (2013)	Partially	Not applicable	No	Yes	Yes	Yes	Cannot tell	Cannot tell	Yes	Yes
Richardson et al. (2023)	Partially	No	No	Partially	Yes	Yes	Cannot tell	Cannot tell	Partially	Yes
Scheunemann et al. (2019)	Yes	Not applicable	Cannot tell	Partially	Yes	Yes	Yes	Yes	No	Yes

### Information analysis

Findings of the studies were reported according to the measure of psychotic experience used, which included broad measures of overall psychotic experiences, measures focusing on voice‐hearing, measures capturing unusual beliefs and measures of paranoid ideation. It should be noted that whilst there are overlaps between paranoia (persistent feelings or thoughts that one is being threatened or persecuted; Freeman & Loe, 2023) and unusual beliefs (fixed beliefs based on inaccurate interpretations of an external reality despite contradictory evidence; Joseph & Siddiqui, 2023), there are distinctions between conceptualisation and measurements of these experiences. It has been argued that a spectrum of severity of paranoia exists, and the severest form is often referred to as ‘persecutory delusions’, where paranoid ideas are held with strong conviction and great distress (Freeman, 2016). However, not all unusual beliefs involve paranoid ideas, and therefore studies investigating unusual beliefs will be discussed separately.

## RESULTS

### Study characteristics

18 studies were included in the review, with both clinical (*N* = 8), non‐clinical (*N* = 7) and mixed clinical and non‐clinical samples (*N* = 2). Eight studies used global measures of positive and negative symptoms of psychosis. Seven studies used hallucination‐specific measures, five studies utilised delusion‐specific measures and four studies measured paranoia.

Nine studies measured self‐compassion, six studies measured self‐criticism and two studies measured both self‐compassion and self‐criticism. All studies that looked at self‐compassion used the SCS, with five using the short form (Raes et al., 2011). Four studies used the one‐factor model, three studies used both the one‐factor and six‐factor model, three used the two‐factor model and one used the six‐factor model (see Table 3). Of the seven studies that looked at self‐criticism, the majority used the FSCRS (*N* = 7), whilst one study used the self‐hate subscale of the FSCRS. Four studies also used the FSCS.

The majority of studies employed cross‐sectional designs (*N* = 15), whilst two studies used a case–control design (Hutton et al., 2013; Richardson et al., 2023). Sample sizes ranged from 49 (Hutton et al., 2013) to 2065 participants (Cheli et al., 2023). Mean age of samples ranged from 16.0 to 42.29, and samples included both men and women, though for most studies (*N* = 9) there was a higher proportion of female participants. The majority of studies did not provide information on the ethnicity of participants (*N* = 10). Of the studies that did report ethnicity, four studies' samples were predominantly comprised of participants who identified as White and two study samples were predominantly comprised of participants who identified as African American (Eicher et al., 2013; Hochheiser et al., 2020).

### Risk of bias

The results of the Risk of Bias Assessment are reported in Table 4. Overall, the quality assessment indicated the studies were of high quality. In particular, most studies clearly defined their inclusion and exclusion criteria and study settings, used valid and reliable measures of psychotic experiences, self‐criticism and self‐compassion and used appropriate statistical analysis methods. Studies used a range of measures to capture psychosis experiences, including both clinician‐administered and self‐report measures.

The most common methodological problems were the representativeness of samples and control of confounding variables, particularly for gender and education. Several studies had a much higher proportion of female participants, yet did not control for gender in the analysis. Considering that gender has been found to be a significant predictor of negative self‐compassion (Hochheiser et al., 2020) and that a meta‐analysis found that females scored lower than males on all six subscales of the SCS (Yarnell et al., 2015), gender may have had a confounding effect on findings. Similarly, several studies did not control for education or ethnicity in their analysis, despite lower levels of education being associated with a higher risk of psychosis (Ayesa‐Arriola et al., 2023), and a higher incidence of psychosis amongst black and other ethnic minority groups (Oduola et al., 2021).

The quality appraisal informed how much weight was given to each study in drawing conclusions. During synthesis, lower‐quality studies were not excluded, but instead a weight‐of‐evidence approach was adopted (Gough, 2007).

### Are self‐compassion and self‐criticism related to overall positive and negative symptoms of psychosis/psychotic‐like experiences?

Three studies examined the link between self‐compassion and positive and negative symptoms of psychosis (Eicher et al., 2013; Hochheiser et al., 2020; Richardson et al., 2023). Two studies investigated self‐compassion and negative symptoms only (Böge et al., 2022; Hardman et al., 2023), and one study examined the relationship between self‐criticism and psychotic‐like experiences (Collman et al., 2023).

Findings indicate that higher total self‐compassion is generally associated with lower severity of positive symptoms in both clinical and non‐clinical samples, though evidence utilising multi‐factor measures of self‐compassion indicated that the negative components (e.g., self‐judgement, isolation, over‐identification) were more strongly and reliably related to symptom severity and associated distress. Importantly, Eicher et al. (2013) found varying relationships between positive and negative aspects of self‐compassion and the positive symptoms of psychosis.

This differential pattern was observed across both clinical and non‐clinical samples. One study reported bivariate associations for both positive and negative self‐compassion with positive symptoms (Hochheiser et al., 2020), but these did not remain significant in regression analyses, suggesting limited independent predictive value. In the same study, sex was associated with negative self‐compassion, indicating a potential confounding effect.

Only one study examining self‐criticism (Collman et al., 2023) indicated a positive association between the severity of positive and negative symptoms and self‐hating and self‐inadequate forms of self‐criticism. However, self‐hating emerged as the only significant predictor of psychotic symptoms.

Overall, the evidence suggests that self‐compassion and self‐criticism are associated with the presence and severity of positive symptoms in clinical and non‐clinical populations, with evidence indicating that self‐criticism (captured by the FSCRS, two‐factor and six‐factor SCS) may be more strongly associated with psychotic experiences than self‐compassion. The evidence was more limited for negative symptoms, though again the emerging picture is that negative self‐to‐self relating is more strongly associated with symptoms than positive self‐to‐self relating.

#### Are self‐compassion and self‐criticism related to voice‐hearing?

Four studies examined the link between self‐compassion and hallucinatory experiences (Dudley et al., 2018; Maisey et al., 2022; Norman et al., 2020; Scheunemann et al., 2019) whilst one study measured self‐criticism (Connor & Birchwood, 2013). The majority of studies specifically focused on voice‐hearing, as opposed to other types of experiences such as visual hallucinations.

The findings demonstrate a consistent inverse association between levels of self‐compassion and distress related to hallucinatory experiences. All studies found that higher total self‐compassion was associated with lower levels of voice‐related distress. In contrast, findings for voice frequency and severity were less consistent, with only one study demonstrating a significant inverse association between self‐compassion and severity and frequency of voice hearing, and others reporting no relationship. This suggests self‐compassion may be more closely related to the emotional impact of voices than to their occurrence or intensity.

Studies examining multidimensional models of self‐compassion in relation to the distress caused by hallucinatory experiences also indicated a differential pattern between positive and negative components. Negative facets (self‐judgement, isolation and over‐identification) were more strongly and consistently associated with voice‐related distress than positive facets. In particular, over‐identification and isolation showed the strongest relationships with distress, and lower self‐compassion was more strongly linked to distress than to the frequency of hallucinatory experiences.

Only one study examined self‐criticism in relation to voice‐hearing (Connor & Birchwood, 2013). They found that self‐critical feelings of inadequacy were associated with greater negative emotional expression of voices, while self‐hating self‐criticism was linked to greater perceived voice power. More persecutory forms of self‐criticism were related to reduced affiliative voice content, and lower self‐reassurance was associated with more shaming voice content. These findings suggest that different forms of self‐criticism may map onto distinct interpersonal and emotional qualities of voices.

Overall, the evidence suggests that self‐compassion is more strongly associated with voice distress than with frequency or severity and that the negative facets of self‐compassion are more strongly related to voice distress than the positive aspects of self‐compassion. Self‐criticism related to inadequacy appears to be positively associated with the emotional expression of voices, whilst self‐hating self‐criticism appears to be positively associated with the perceived power of the voices.

#### Are self‐compassion and self‐criticism related to unusual beliefs?

One study looked at the link between self‐compassion and the severity and frequency of unusual beliefs (Scheunemann et al., 2019), whilst two studies examined the relationship between self‐criticism and the severity of unusual beliefs (Cheli et al., 2023; Kingston & Schuurmans‐Stekhoven, 2016).

Across studies, a consistent pattern emerged, linking negative self‐relating processes with greater severity of unusual beliefs. Both studies examining self‐criticism reported positive associations between self‐criticism and the severity of unusual beliefs, alongside evidence that self‐reassurance may be protective. However, the studies did not explore whether different forms of self‐criticism (self‐hating vs. self‐inadequate) might differentially relate to unusual beliefs. Cheli et al. (2023) found that self‐hating self‐criticism moderated the relationship between impaired self‐reflectivity and delusional ideation, suggesting that harsh self‐evaluation may exacerbate the effects of broader metacognitive difficulties.

Evidence examining self‐compassion indicates that both the overall level and specific components of self‐compassion may be associated with unusual beliefs. Scheunemann et al. (2019) found that greater distress, frequency, preoccupation and conviction associated with unusual beliefs were linked to lower overall self‐compassion and lower scores on the positive self‐compassion subscales, while being positively associated with the negative self‐compassion components. Notably, negative self‐relating appeared to be more strongly associated with the distress linked to unusual beliefs than with their frequency, suggesting that self‐critical or hostile self‐evaluative processes may play a particularly important role in the emotional impact of psychotic‐like experiences.

Overall, the evidence suggests that negative self‐to‐self relating (captured by negative items of SCS and the FSCS/FSCRS) is associated with greater delusional ideation. However, only one study distinguished between frequency and distress of unusual beliefs, finding that total‐self compassion and the negative SCS items were more strongly related to distress than frequency. It should also be noted that studies did not examine differences in the form (inadequate‐self vs. hated‐self) and function of self‐criticism (self‐persecutory vs. self‐corrective).

#### Are self‐compassion and self‐criticism related to paranoia?

One study examined the relationship between paranoia, self‐compassion and self‐criticism (Mills et al., 2007) and three studies examined the link between paranoia and self‐criticism only (Carvalho et al., 2019; Hutton et al., 2013; Pinto‐Gouveia et al., 2013).

Across studies, a consistent pattern emerged linking negative self‐relating (particularly self‐persecutory self‐criticism) with higher levels of paranoia in both clinical and non‐clinical populations. Overall, the findings suggest that paranoia is more strongly associated with self‐critical processes than with positive self‐to‐self relating, such as self‐reassurance or compassionate self‐responding. Importantly, studies varied in whether they independently examined different forms and functions of self‐criticism, with one study not distinguishing between either form or function (Carvalho et al., 2019).

The evidence indicated that self‐hating and self‐persecutory self‐criticism may be particularly relevant to paranoia. For example, Mills et al. (2007) found self‐hating criticism emerged as the strongest predictor of paranoia compared to the negative components of self‐compassion, self‐inadequacy and self‐persecution. Findings from other studies also indicated that self‐criticism with a self‐persecutory function was strongly linked to paranoia and that self‐corrective self‐criticism was not related (Hutton et al., 2013; Pinto‐Gouveia et al., 2013). However, the findings of Hutton et al. (2013) should be interpreted cautiously due to methodological limitations, including a small sample size and between‐group differences in age and education, particularly as age has been negatively associated with self‐attacking (Gilbert et al., 2004).

Taken together, the evidence suggests that self‐hating and self‐persecutory forms of self‐criticism may play a particularly important role in paranoia, whereas self‐corrective self‐criticism and positive self‐relating appear less strongly associated. However, the literature remains limited by inconsistent measurement approaches and a lack of differentiation between forms and functions of self‐criticism, constraining conclusions about the specific mechanisms linking self‐relating processes and paranoid ideation.

## DISCUSSION

### Summary of findings

This paper provides a systematic review of the literature investigating the relationship between self‐compassion, self‐criticism and psychotic experiences in clinical and non‐clinical populations. Self‐compassion and self‐criticism appear to be associated with a range of different psychotic experiences, including positive and negative symptoms, voice‐hearing, delusional beliefs and paranoia. Findings indicate that negative aspects of self‐compassion and self‐criticism may be more strongly linked to psychotic experiences than positive aspects of self‐compassion. Furthermore, self‐attacking forms of criticism with a self‐persecutory function may be more potent than self‐inadequate self‐criticism with a corrective function. Evidence also indicates that self‐criticism and self‐compassion may be more strongly linked to the distress associated with psychotic experiences rather than the frequency or severity of symptoms.

### Self‐criticism, self‐compassion and broad symptom measures: Overlooking nuances?

Whilst there was strong evidence to suggest that lower levels of self‐compassion and higher levels of self‐criticism are associated with the presence and severity of positive symptoms in clinical and non‐clinical populations, one study (Eicher et al., 2013) highlighted that using broad symptom measures may not adequately capture how nuances in self‐to‐self relating might be related to specific areas of psychotic experiences (e.g. voice‐hearing, unusual beliefs, paranoia).

The findings suggest that more extreme self‐attacking forms of self‐criticism with a self‐persecutory function, as opposed to feelings of inadequacy and wanting to improve, may be more strongly related to the severity of positive symptoms and levels of paranoia. This suggests that active negative self‐to‐self relating may represent a risk factor for psychotic experiences, more so than just an absence of self‐compassion. Considering research that shows that increasing self‐compassion reduces levels of self‐criticism (Wakelin et al., 2022), perhaps self‐compassion could be considered a resilience factor for psychotic experiences and distress via its effect on self‐criticism.

These findings highlight potential problems in the use of measures that do not distinguish between the positive and negative aspects of self‐compassion and self‐criticism. Previous research has indicated that the negatively worded items of the SCS are components of self‐criticism and so a two‐factor model should be used (Ferrari et al., 2022; López et al., 2015; Muris & Petrocchi, 2017), whilst others have argued for the use of a six‐factor SCS to provide a more granular and nuanced insight into the nature of self‐to‐self relating and links with psychopathology (Castilho et al., 2015; Cleare et al., 2018; Kumlander et al., 2018). Thus, overall measures of self‐compassion may not capture nuances in the relationship between self‐criticism, self‐compassion and psychotic experiences.

### Voice‐hearing and unusual beliefs – A matter of distress?

Negative aspects of self‐compassion, specifically isolation and overidentification, appeared to be more closely related to voice and hallucination distress than frequency, suggesting that fixating on and feeling alone with one's problems play a pivotal role in distress. This could also be linked to difficulties with finding balanced perspectives on one's experiences, such as perseverative thinking (Černis et al., 2016), rumination (McKie et al., 2017) and worry (Hartley et al., 2014) which have all been associated with psychosis. The association between isolation and distress with psychotic‐like experiences is also in line with research on the high prevalence of loneliness and isolation in psychosis (McCarthy‐Jones et al., 2016; Michalska da Rocha et al., 2018).

Together, the findings support theories on the importance of how individuals relate to themselves and their psychotic experiences. For example, Social Rank Theory (Gilbert, 2001) suggests that voice malevolence or persecutory beliefs could be received as a threatening social cue, activating the threat system (i.e., stress‐cortisol), resulting in distress. Distress may then inhibit feelings of self‐compassion, aligning with evolutionary theories that when in a state of threat, people are less likely to access their ‘soothing system’ as this would enhance vulnerability to attack (Gilbert, 2009). In contrast, developing compassionate ways of relating to oneself and voices may influence a less threatening perception of social rank and subsequently less distress (Paulik, 2012). The link between self‐criticism and distress is particularly important, as studies have found that distress is one of the most significant factors distinguishing people with and without a diagnosis of psychosis (Lincoln, 2007).

### Self‐criticism and paranoia

There was strong evidence to indicate that self‐criticism was associated with paranoid ideation. Several studies identified that self‐persecutory forms of self‐criticism were most strongly related to paranoia (Hutton et al., 2013; Mills et al., 2007; Pinto‐Gouveia et al., 2013), supporting the idea that self‐criticism with a self‐corrective function can be adaptive, whilst self‐persecutory self‐criticism (where people criticise themselves in order to destroy or take revenge on themselves) may be more closely linked to emotional distress (Gilbert et al., 2004).

Two studies also found correlations between shame, self‐criticism and paranoia (Carvalho et al., 2019; Pinto‐Gouveia et al., 2013) aligning with the social rank theory that argues that shame experiences (e.g., being criticised, shamed, abused, or rejected) may result in negative internal working models about the self (e.g., self as defective or inferior) which in turn increase vulnerability to self‐criticism as an emotional self‐regulation strategy (Castilho et al., 2017; Gilbert & Procter, 2006; Pinto‐Gouveia & Matos, 2011). Thus, paranoia and self‐criticism may represent defensive strategies against perceived threats from others, though in the case of self‐criticism the hostility is experienced internally (Gilbert et al., 2001; Gilbert & Procter, 2006).

### Strengths, limitations and future research

A strength of this review is that a relatively high number of good‐quality studies were included. Several studies included large sample sizes and both clinical and non‐clinical populations, allowing for examination of co‐variation between psychotic experiences, self‐criticism and self‐compassion based on the full continuum of experiences. However, it should be noted that including clinical and non‐clinical samples may increase heterogeneity in how psychotic experiences are conceptualised and measured, and thus, findings should be interpreted with caution (Hinterbuchinger & Mossaheb, 2021). It was beyond the scope of this review to statistically explore differences in the relationship between self‐criticism, self‐compassion and psychotic experiences between these two populations, but this could be explored further in future research.

Studies varied in their measures of psychosis, use of overall or multi‐factorial measures of self‐compassion and examination of different forms and functions of self‐criticism. Whilst it was beyond the scope of the current review, future research could clarify quantitative differences in the relationships between different measures of self‐criticism, self‐compassion and psychotic experiences. This would add to the findings of this review, which tentatively suggest the use of the one‐factor SCS may be problematic, and indicate the importance of distinguishing between positive and negative aspects of self‐compassion and self‐criticism in future research. Given neurobiological research indicating differing neural pathways are involved in self‐compassion and self‐criticism (Kim et al., 2020), further fMRI studies and elucidation of potential biomarkers may also aid in the conceptualisation and measurement of self‐compassion and self‐criticism and how these relate to psychotic experiences.

As the evidence indicates that self‐criticism and self‐compassion may be more strongly linked to distress associated with psychotic experiences, it would be helpful for future research to use distinct measures of distress, frequency and severity to allow for further statistical comparisons. Since distress has been highlighted as a distinguishing factor between people with and without diagnosable psychosis (Lincoln, 2007), further research using case–control designs could explore differences in self‐compassion, self‐criticism and distress associated with psychotic experiences between clinical and non‐clinical samples. Furthermore, studies are mainly focused on voice‐hearing as opposed to other hallucinatory experiences (e.g., visual, tactile). This is an important gap in the current research, given evidence indicating a link between trauma, self‐criticism and vulnerability to voice‐hearing (Maisey et al., 2022; Norman et al., 2020) as well as studies suggesting that self‐criticism and threat‐related processes may overlap with experiences such as dissociation, somatic sensations and sensory intrusions (Gilbert, 2022; Krause‐Utz et al., 2017). Future research could also consider other factors associated with both psychotic experiences and self‐criticism, such as shame and general psychopathology, such as anxiety, depression and stress (Carden et al., 2020; Castilho et al., 2015), and how they may interact.

The cross‐sectional and observational nature of the studies included precluded conclusions about causation and direction of the relationship between self‐compassion, self‐criticism and psychotic experiences. However, other authors have provided evidence that cultivating self‐compassion over time supports recovery in psychosis (Mavituna et al., 2023). Longitudinal research designs could provide insight into self‐criticism and self‐compassion as risk or resilience factors in the development of psychosis, for example, using a repeated measures design to capture changes in self‐compassion, self‐criticism and severity, distress and endorsement of psychotic experiences in high‐risk individuals.

### Clinical implications

High levels of self‐criticism, shame and stigma, perhaps driven by a perception of self as subordinate (Gilbert et al., 2001), may mean that engaging in therapies feels more threatening for people with psychosis. This is supported by a review that found that feelings of disempowerment, blame and stigma are barriers to engaging with services (Berry & Haddock, 2008). Importantly, self‐criticism and a perceived subordinate role in therapy have been found to be associated with greater difficulty in establishing and maintaining a therapeutic alliance (Whelton et al., 2007), which is understood as a collaboration between therapist and client within the context of a secure relationship (Bordin, 1979). Thus, therapeutic interventions with people with psychosis may need greater time spent building trust, security and relational safety.

Considering research indicating that distress is the most important distinguishing factor between people with and without a diagnosis of psychosis (Lincoln, 2007) and that self‐criticism and self‐compassion appear to be strongly linked to distress, perhaps these should be more centrally focused outcomes for psychological interventions. For example, Compassion Focused Therapy (CFT) for psychosis (Gilbert, 2009) was developed in response to the observation that shame and self‐criticism could present therapeutic barriers in traditional Cognitive Behavioural Therapy (Gilbert, 2000, 2009). CFTp aims to reduce shame and self‐criticism by developing self‐compassion through psychoeducation, imagery and mindfulness (Braehler et al., 2013; Gilbert, 2014). A systematic review (Mavituna et al., 2023) found good acceptability and feasibility of compassion‐based interventions for psychosis, with lower levels of self‐criticism appearing to drive improvements in mood and self‐esteem, and reduction in positive and negative symptoms. Recent research has also highlighted that CFTp is feasible and acceptable, demonstrating improvements in depression, stress, distress and severity of voices and delusions (Heriot‐Maitland et al., 2023; Heriot‐Maitland et al., 2025); although further high‐quality research is needed to add to the emerging evidence base. This review suggests that self‐criticism and self‐compassion could potentially be important mechanisms to be addressed by psychological therapies and warrants further research attention.

## CONCLUSION

Considering the high levels of self‐criticism and difficulties with self‐compassion that are apparent in people with psychotic experiences, this review aimed to synthesise the current evidence base regarding the relationship between self‐criticism, self‐compassion and psychotic experiences. Overall, the evidence indicates that self‐criticism may be more strongly associated with positive symptoms in both clinical and non‐clinical populations than the positive aspects of self‐compassion. The evidence suggests that self‐compassion and self‐criticism may be more strongly associated with the distress accompanying experiences of voice‐hearing and unusual beliefs, rather than their severity or frequency. Findings also indicated that self‐persecutory self‐hating forms of self‐criticism were strongly linked to paranoid ideation. However, as a meta‐analysis was not conducted, it was not possible to statistically compare relationships between self‐compassion, self‐criticism and different areas of psychotic experiences, which is an important area for future research. Moreover, as all included studies were cross‐sectional, this precludes conclusions about the direction and causality of relationships. It is important to consider the potential influence of confounding demographic and other variables, such as shame and general psychopathology. Nonetheless, the findings indicate that self‐criticism and self‐compassion may represent risk and resilience factors in psychotic experiences and associated distress that could be addressed through compassion‐based psychological therapies.

## AUTHOR CONTRIBUTIONS

**Megan Crawford:** Conceptualization; formal analysis; project administration; visualization; writing – original draft; writing – review and editing. **Rachel Terry:** Conceptualization; supervision; writing – original draft; writing – review and editing. **Leila Jameel:** Conceptualization; supervision; writing – original draft; writing – review and editing.

## CONFLICT OF INTEREST STATEMENT

The authors declare no conflicts of interest.

## Data Availability

The data that support the findings of this study are available on request from the corresponding author. The data are not publicly available due to privacy restrictions.
